# Altered Mental Status in the Setting of Thrombotic Thrombocytopenic Purpura (TTP) and Spontaneous Coronary Artery Dissection (SCAD): A Case Report and Literature Review

**DOI:** 10.7759/cureus.54642

**Published:** 2024-02-21

**Authors:** Esmirna Perez, Nehemias Guevara, Jordan Smith, Ricardo Velasquez

**Affiliations:** 1 Medicine, St. Barnabas Hospital Health System, New York, USA; 2 Critical Care Medicine, St. Barnabas Hospital Health System, New York, USA

**Keywords:** acute coronary syndrome (acs), spontaneous coronary artery dissection (scad), microangiopathic hemolytic anemia (maha), thrombotic thrombocytopenic purpura (ttp), altered mental status

## Abstract

Altered mental status (AMS) is a common condition encountered in daily practice. Finding the cause is essential for treatment, but sometimes this may be challenging. Spontaneous coronary artery dissection (SCAD) is frequently underdiagnosed and is a potentially fatal cause of acute coronary syndrome. Clinical presentation depends on the extent of SCAD, ranging from unstable angina to sudden death. AMS has not been reported with this condition, but it may be possible in hypoperfusion states.

Thrombotic thrombocytopenic purpura (TTP) is part of the microangiopathic hemolytic anemia (MAHA) spectrum, presenting with AMS as the cardinal symptom. TTP is a clinical emergency, and a high index of suspicion should be present as the mortality rate in untreated patients is extremely high and can be significantly reduced with proper treatment.

We present a case of a 44-year-old female with a past medical history of antiphospholipid syndrome not on anticoagulation, peptic ulcer disease, chronic kidney disease, stroke, seizures, congestive heart failure with reduced ejection fraction (EF 40%), two non-ST-segment elevation myocardial infarctions not on dual antiplatelet therapy due to a history of gastrointestinal bleeding, and TTP, admitted to the hospital with AMS. The patient was diagnosed with two life-threatening pathologies with overlapping features but opposing management; TTP may have been caused by SCAD, even though this has never been reported. It is essential to recognize that while a single diagnosis frequently explains a patient's clinical manifestations, there are instances when various conditions may be present.

## Introduction

Altered mental status (AMS) is a broad term frequently used to describe any change in a patient's baseline state of awareness. This is the presenting sign for up to 5-10% of patients visiting the emergency department (ED). These changes can have different onset times, being classified as acute, subacute, or chronic. Acute changes can present in minutes to days, can be life-threatening, and are often secondary to an acute medical condition [1].

Multiple factors that increase the vulnerability of patients to develop AMS have been recognized, such as older age, dementia, functional impairment, multiple comorbidities, and substance abuse. The presence or absence of these factors aids in the recognition of the severity at presentation [1]. Initial assessment of any patient with AMS should prioritize airway, breathing, and circulation. Once these parameters are checked, the presence of immediately life-threatening conditions, such as hypo- or hyper-glycemia, drug intoxications, myocardial infarction, stroke, sepsis, or seizures, must be identified and corrected. In these scenarios, immediate treatment is required to prevent further adverse events [2].

We present a case of a 44-year-old female with an extensive medical history who presented with AMS and was found to have two life-threatening diseases and their management. To our knowledge, this is the first case ever of these deadly diseases presenting together. 

## Case presentation

A 44-year-old female with a past medical history of antiphospholipid syndrome not on anticoagulation, peptic ulcer disease, chronic kidney disease, stroke, seizure disorder, congestive heart failure with a reduced ejection fraction of 40%, two non-ST-segment elevation myocardial infarctions not on dual antiplatelet therapy due to history of gastrointestinal bleeding, and thrombotic thrombocytopenic purpura (TTP) was admitted to the hospital with AMS. 

She initially presented to the ED complaining of one day of constant abdominal pain associated with shortness of breath. Upon admission, she had an elevated blood pressure of 186/100 mmHg, was tachypneic with a respiratory rate of 23 breaths per minute, had a heart rate of 88 beats per minute, and had AMS with a Glasgow Coma Scale (GCS) of 8. Capillary blood glucose level was 22 mg/dL. After initial treatment with intravenous dextrose infusion, there was no improvement in mental status. She was then intubated for airway protection.

Initially, diagnostic tests (Table 1) were remarkable for elevated creatinine, transaminitis, hyperbilirubinemia, anemia, and thrombocytopenia. EKG showed new ST-segment elevation in leads II and III, and serum troponin enzyme was elevated; therefore, cardiac catheterization was performed.

**Table 1:** Initial workup MCV: mean corpuscular volume; BUN: blood urea nitrogen; ALT: alanine transaminase; AST: aspartate aminotransferase; INR: international normalized ratio; LDH: lactate dehydrogenase

Variable	On admission	Reference range
White blood cell count	14.5	4.0-10.0 x 10^3^/uL
Hemoglobin	10	11.2-15.7 gm/dL
Hematocrit	33	34.1-44.9%
MCV	80.7	79.4-94.8 fL
Platelet count	72	150-450 x 10^3^/uL
Reticulocyte	4.73	0.50-1.70%
Immature reticulocyte	38.3	3.0-15.9%
Reticulocyte absolute	0.1253	0.0164-0.0776
Creatinine	5.2	0.6-1.2 mg/dL
BUN	71	8-23 mg/dL
ALT	494	4-36 IU/L
AST	434	8-33 IU/L
Bilirubin total	3.3	0.1-1.2 mg/dL
INR	1.5	0.9-1.1
Calcium	9	9.2-11.0 mg/dL
Albumin	2.8	3.8-5.0 gm/dL
LDH	602	100-190 IU/L
Troponin	5.05	0.00-0.48 ng/mL

**Figure 1:**
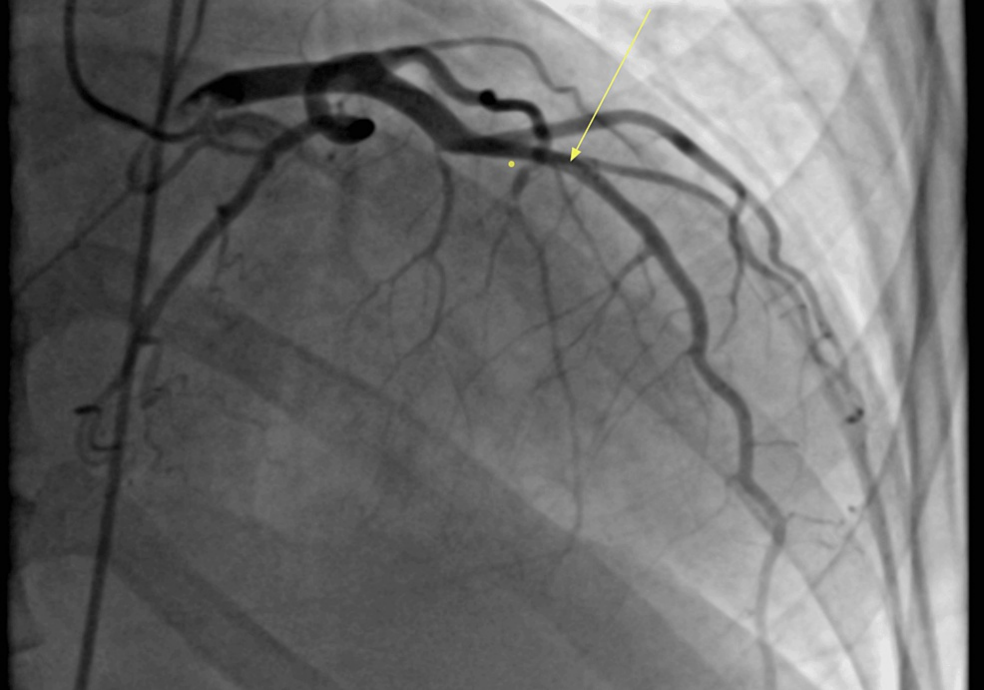
Cardiac catheterization image with apical LAD artery subtotal occlusion (yellow arrow)

**Figure 2:**
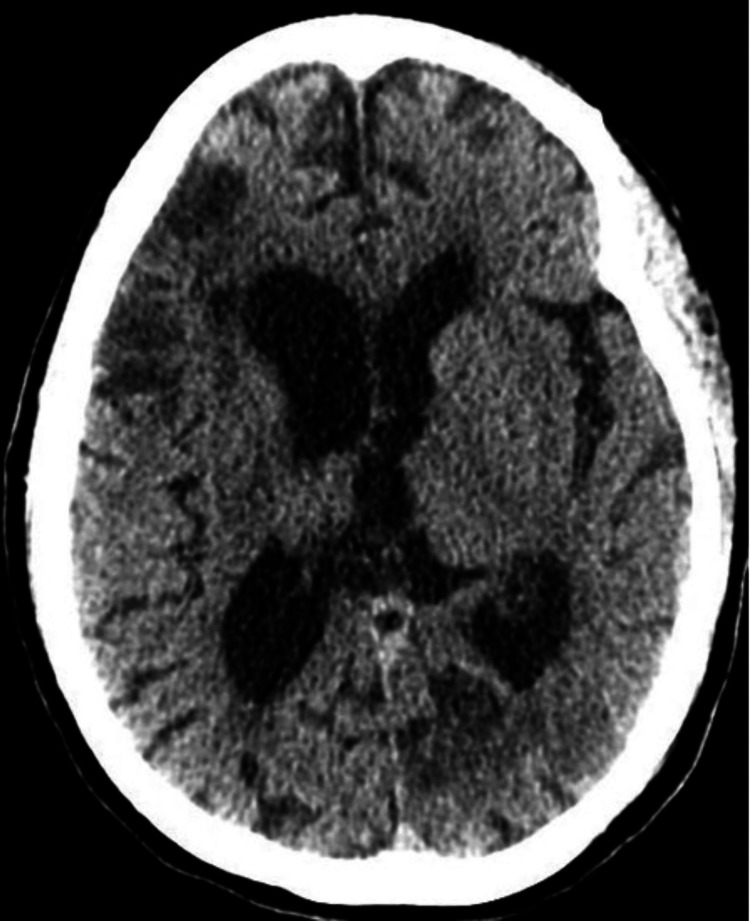
Brain atrophy with chronic large and small vessel ischemic changes and no evidence of acute infarct

During the cardiac catheterization procedure, apical left anterior descending (LAD) artery subtotal occlusion was seen, described as spontaneous coronary artery dissection (SCAD) (Figure 1). The patient was started on heparin infusion and was weaned off sedation without improvement in mental status. Computed tomography (CT) head ruled out an acute intracranial pathology (Figure 2). Platelets further decreased on the second day of admission to 50,000, and heparin infusion was stopped as a suspected cause of thrombocytopenia. A workup for heparin-induced thrombocytopenia (HIT) was sent. However, the HIT expert probability (HEP) score of -1 suggested a low probability of HIT. Other causes of thrombocytopenia with hemolytic anemia were contemplated.

Lactate dehydrogenase (LDH) increased with progressively worsening anemia and renal function. Therefore, concomitant microangiopathic hemolytic anemia (MAHA) was considered. The peripheral blood smear showed schistocytes, and the patient was treated with plasmapheresis and high-dose steroids at a dose of 1 mg/kg for a total of 10 days for suspected TTP. PLASMIC (Platelet count, combined hemoLysis variable, absence of Active cancer, absence of Stem cell or solid-organ trasplant, MCV, INR, Creatinine) score was 5; it was considered an intermediate risk for TTP. The diagnosis was confirmed afterward with decreased activity of a disintegrin and metalloproteinase with a thrombospondin type 1 motif, member 13 (ADAMTS13) and an elevated ADAMTS13 antibody. After plasmapheresis, the patient's mental status improved, and she was successfully extubated. Clinical improvement happened with associated LDH level reduction and platelet count increase. She is currently following up with hematology-oncology as an outpatient without any residual deficit and complete resolution of laboratory abnormalities.

## Discussion

Changes in patients’ baseline level of consciousness, defined as AMS, is a common presenting sign in critically ill patients. It can be the initial clinical sign of significant cerebral hypoperfusion in conditions that compromise the heart’s ability to circulate blood, and therefore oxygen, to vital organs. Because of this relationship, it has been defined as one of the diagnostic criteria for cardiogenic shock. AMS in the setting of an acute coronary syndrome (ACS) has been shown to be instantaneous with an appreciable drop in blood pressure as opposed to hypoperfusion seen in cardiogenic shock in the setting of, for example, chronic decompensated heart failure. Studies have shown that mental status assessment has prognostic significance in cardiogenic shock. AMS has been associated with prolonged hospital stays and increased mortality in patients with compromised cardiac function [3,4].

SCAD is frequently underdiagnosed and described as a potentially fatal cause of ACS. SCAD is a spontaneous, non-traumatic, and nonatherosclerotic separation of the coronary artery wall due to an intramural hemorrhage. Clinical presentation depends on the extent of the separation, ranging from unstable angina to sudden death. It presents most commonly in women between the ages of 42 and 53 years and is the most common cause of myocardial infarction related to pregnancy. Myocardial infarction is the initial manifestation of SCAD in more than 90% of patients, leading in some cases to ventricular arrhythmias and cardiogenic shock. Chest pain is the patients' most frequent main complaint, with or without dyspnea and diaphoresis [5,6].

Diagnosis is made by coronary angiography, showing a separation of the coronary artery wall. In instances where said diagnosis is uncertain even after angiography, other imaging techniques can be utilized for confirmation, such as intravascular ultrasonography (IVUS) and optical coherence tomography (OCT). Nevertheless, up to 8% of patients have developed complications after intravascular imaging, such as extension of the dissection, iatrogenic dissection, etc. Other ancillary imaging modalities, like coronary computed tomographic angiography (CCTA), provide a limited visualization with the consequent risk of false negative results. For all the aforementioned reasons, coronary angiography continues to be the diagnostic standard [5,6].

The etiology of the disease is still not fully elucidated. However, it has often been associated with fibromuscular dysplasia (FMD), emotional or physical stress (Valsalva, retching, coughing, exercise), stimulant medications or illicit drugs, hormonal triggers (pregnancy), inflammatory diseases, and hypertension [5].

There are no current guidelines on the specific treatment of SCAD, but conservative treatment with medical management similar to other causes of ACS has been recommended. Medical management includes antiplatelet drugs, intravenous heparin, and beta blockers. In one meta-analysis performed utilizing observational studies data, there was no difference in rates of death, recurrence, or revascularization in patients treated with medical therapy when compared with those treated with invasive therapy (percutaneous coronary intervention (PCI)) [7,8]. Nevertheless, PCI can be challenging in these patients and associated with serious complications as is coronary stent embolization, the instance wherein the stent placed travels through the coronary circulation [9]. SCAD has been seen to resolve in 73-97% of cases spontaneously, vastly supporting the conservative approach [5].

Our patient presented with AMS upon admission, and though her hypoglycemia could have explained this, there was no improvement with appropriate treatment. Furthermore, ACS-induced cardiogenic shock did not seem a plausible cause as our patient was hemodynamically stable and had no other findings commonly found in hypoperfused states, such as lactic acidosis or oliguria. Other triggering pathologies had to be considered. 

TTP is part of the thrombotic microangiopathy (TMA) spectrum and is a life-threatening angiopathy secondary to a severe deficiency of ADAMTS13. ADAMTS13 is a specific von Willebrand factor (VWF) cleaving protease produced by hepatic stellate cells, podocytes, renal tubular epithelial cells, platelets, and endothelial cells. VWF are multimers with an ultra-large structure secreted by the endothelial cells from the Weibel-Palade bodies and stay bound to the endothelium through protein interactions. The conformation of VWF changes with arterial shear stress, making the ADAMTS13 cleavage domain accessible [10].

The most commonly affected organ at presentation is the nervous system (up to 40-80% of cases). Neurological signs and symptoms can include AMS, headache, coma, seizure, stroke, and/or reversible focal deficits. A GCS of 14 or less was associated with a ninefold increase in mortality compared to patients with a normal score of 15. Other symptoms commonly seen in the acute phase are gastrointestinal symptoms and cardiac and renal disorders [11].

Cardiac manifestations of TTP can include chest pain, heart failure symptoms, and EKG changes [12]. Elevated troponin has been reported in 68% of patients [11]. Severe cardiac involvement has been described with the presence of cardiomyopathy, myocardial infarction, and even sudden cardiac death. All symptoms of TTP are secondary to organ ischemia caused by microvascular platelet-rich thrombi. Specific signs of TTP are MAHA and severe thrombocytopenia. MAHA can be identified by the presence of schistocytes on a peripheral blood smear, which is caused by the shearing of the red blood cells as they pass through microvascular thrombi [10].

TTP is a clinical emergency, and a high index of suspicion should be present as the mortality rate in untreated patients is as high as 90% and can be reduced to 10-20% with proper treatment. Diagnosis, therefore, should be suspected in all patients with MAHA and thrombocytopenia [13].

Prediction scores incorporating clinical and laboratory data, such as PLASMIC, French, and Bentley, are available to help determine the probability of suspected TTP while waiting for laboratory-confirmed ADAMTS13 deficiency [11].

First-line treatment is daily therapeutic plasma exchange therapy and should be started immediately when TTP is suspected, even if the diagnosis is yet to be confirmed. As the etiology is known to be autoimmune, empiric steroid therapy is used as an adjunct to plasma exchange. Rituximab, a monoclonal antibody against cluster of differentiation 20 (CD20), is commonly used in the acute phase of TTP [10]. Other immunosuppressive therapies often used for refractory disease include cyclosporine A, mycophenolate mofetil, vincristine, bortezomib, and cyclophosphamide. Response to treatment is defined as normal platelet counts for two consecutive days, normalization of low-density lipoprotein (LDL), and clinical improvement [12].

TTP is no longer considered a one-time event, and it has been demonstrated that there are chronic complications of TTP and a risk of relapse in patients who have recovered from the acute episode. How low the ADAMTS13 activity was is related to the risk of recurrence; therefore, the higher relapse rates are in cases where the activity levels are less than 10-20% and 10 times higher in patients with severe deficiency compared to patients with normal levels. Monitoring enzyme activity levels has been recommended to predict and prevent relapse. In some instances, immunosuppressive therapy with rituximab and cyclosporine has been proposed in high-risk patients for recurrence prevention [12,14].

In the present case, SCAD and TTP developed together with overlapping signs and symptoms. Both pathologies can present features such as chest pain, elevated troponin, EKG changes, and altered mental status. Because both entities have a low prevalence, 0.1-4% for SCAD and 2.7 cases per million for TTP, diagnosing our patient was very difficult [5,15].

To our knowledge, a relationship between SCAD and TTP has not been described before. There has, however, been one published report of acute aortic dissection occurring in the setting of TTP. The patient was recovering from an acute TTP attack when he started showing signs and symptoms leading to the diagnosis of an aortic dissection. Like our patient, the described patient had a baseline autoimmune disease and presented with low ADAMTS13 levels and high levels of ADAMTS13 inhibitors [16]. As both medical conditions TTP and SCAD can have some clinical features in common, the relative onset timing cannot be accurately determined. We believe that SCAD might have induced TTP, even though this has not been described before. Further research is required on the presentation and inciting factors of SCAD to determine appropriate evidence-based treatment.

## Conclusions

Our patient represented a challenging diagnosis of AMS. She had two life-threatening pathologies with overlapping features but opposing managements. Her TTP may have been caused by SCAD, even though that has never been reported. It is important to recognize that a single diagnosis often explains a patient's clinical manifestations, but there are instances when multiple conditions may be present.
